# Aryl Hydrocarbon Receptor in Oxidative Stress as a Double Agent and Its Biological and Therapeutic Significance

**DOI:** 10.3390/ijms23126719

**Published:** 2022-06-16

**Authors:** Alevtina Y. Grishanova, Maria L. Perepechaeva

**Affiliations:** Federal Research Center of Fundamental and Translational Medicine, Institute of Molecular Biology and Biophysics, Timakova Str. 2, 630117 Novosibirsk, Russia; agrish@niimbb.ru

**Keywords:** reactive oxygen species, oxidative stress, antioxidant, aryl hydrocarbon receptor, AhR, nuclear factor-erythroid 2-related factor 2, Nrf2

## Abstract

The aryl hydrocarbon receptor (AhR) has long been implicated in the induction of a battery of genes involved in the metabolism of xenobiotics and endogenous compounds. AhR is a ligand-activated transcription factor necessary for the launch of transcriptional responses important in health and disease. In past decades, evidence has accumulated that AhR is associated with the cellular response to oxidative stress, and this property of AhR must be taken into account during investigations into a mechanism of action of xenobiotics that is able to activate AhR or that is susceptible to metabolic activation by enzymes encoded by the genes that are under the control of AhR. In this review, we examine various mechanisms by which AhR takes part in the oxidative-stress response, including antioxidant and prooxidant enzymes and cytochrome P450. We also show that AhR, as a participant in the redox balance and as a modulator of redox signals, is being increasingly studied as a target for a new class of therapeutic compounds and as an explanation for the pathogenesis of some disorders.

## 1. Introduction

In live cells, reactive oxygen species are continuously generated, for example, by xanthine oxidase to degrade purine nucleotides, by nitric oxide synthase to form nitric oxide, and by other biochemical reactions as a byproduct of the oxidative energy metabolism for the formation of adenosine triphosphate from glucose in mitochondria [1,2,3,4]. 

Under normal physiological conditions, small amounts of oxygen are constantly converted into superoxide anions, hydrogen peroxide, and hydroxyl radicals. The biological activity of reactive oxygen species at a physiological concentration plays an important role in cell homeostasis and in a wide range of cellular parameters (proliferation, differentiation, cell cycle, and apoptosis) [5,6,7,8].

In the cell, reactive oxygen species arise under the influence of such exogenous pro-oxidant factors as environmental pollutants, ionizing and ultraviolet radiation, xenobiotics, air pollutants, and heavy metals [9,10].

The main endogenous sites of production of cellular redox-reactive compounds include complexes I and III of the mitochondrial electron transport chain, endoplasmic reticulum, peroxisomes, and such enzymes as membrane-bound nicotinamide adenine dinucleotide phosphate (NADPH) oxidase (NOX) isoforms 1–5 (NOX1–NOX5), complexes of dual oxidases 1 and 2, xanthine oxidase, polyamine and amine oxidases, enzymes catabolizing lipids, and cytochrome P450 family 1 (CYP1A) [11,12,13,14,15,16].

The high reactivity of oxygen and its active species necessitates a multi-level antioxidant defense system that blocks the formation of highly active free radicals [10].

Free radicals are usually eliminated by the body’s natural antioxidant system. Redox homeostasis in normal cells is maintained by a nonenzymatic system consisting of carotenoids, flavonoids, glutathione, anserine, carnosine, homocarnosine, melatonin, thioredoxin, and vitamins C and E, as well as a network of antioxidant enzymes such as superoxide dismutases, catalases, peroxiredoxins, glutathione peroxidase (GPX), glutaredoxins, and paraoxonases [17,18]. In redox homeostasis, a certain role is played by the enzymes of phase II xenobiotic biotransformation, e.g., NADPH:quinone oxidoreductase 1 (NQO1), glutathione-S-transferase (GST) P1, GSTA1/2, UDP glucuronosyltransferase (UGT) 1A6, GPX4, and heme oxygenase 1 [19].

An imbalance between the formation of oxidative free radicals and the antioxidant defense capacity of the body’s cells is defined as oxidative stress. An important function in the regulation of oxidative stress is performed by the AhR signaling pathway via pro-oxidant and antioxidant mechanisms.

## 2. AhR Expression, Functions, and Signaling

### 2.1. AhR Structure

The aryl hydrocarbon receptor (AhR), its partner protein aryl hydrocarbon receptor nuclear translocator (ARNT), and AhR repressor protein (AhRR) are members of a family of structurally related transcription factors (basic helix–loop–helix (bHLH) motif-containing Per–ARNT–Sim (PAS), whose members carry out critical functions in the gene expression networks that underlie many physiological and developmental processes, especially those participating in responses to signals from the environment [20,21]. 

Structurally, human AhR has a sequence of 848 amino acid residues and includes 3 functional domains: from the amino (N-) to carboxy (C-)terminus, these are bHLH, PAS A, PAS B, and transcription activation domains (TADs) whose activity is mediated by coactivators called CBP/p300 and RIP140 [21,22]. 

The amino acid sequence of the bHLH and PAS domains is evolutionarily highly conserved. The bHLH domain can be divided into an HLH domain and a basic domain and is involved in AhR binding to DNA and in protein dimerization [23,24]. The PAS region participates in ligand binding and is thought to be the site of protein–protein interactions during dimer formation; PAS B partially overlaps with the heat shock protein 90 (HSP90)-binding site [21,25]. The transcription activation domain serves as a mediator of the transcriptional activation of downstream genes [26]. 

**Figure 1:**
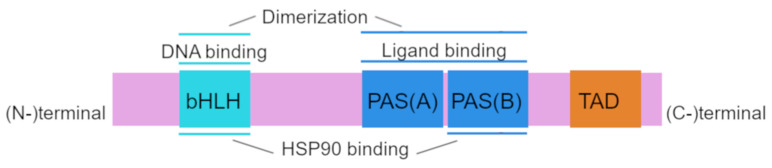
Structure of aryl hydrocarbon receptor (AhR). The basic helix–loop–helix (bHLH) motif, common among various transcription factors, is located at the N terminus of the AhR protein and is involved in DNA binding and protein–protein interactions. Per–ARNT–Sim (PAS) domains (PAS-A and PAS-B) participate in binding to ligands and to HSP90 proteins and in dimerization with partner proteins. The transactivation domain (TAD) is located at the C terminus of the AhR protein.

The AhRR protein is structurally similar to AhR in the bHLH region, and this property allows AhRR to heterodimerize with ARNT and to bind to a xenobiotic-responsive element (XRE) [27]. The repression domain of AhRR contains three sumoylation sites, all of which must be sumoylated for complete repression of AhR target genes [24,28]. Structure of AhR is shown in Figure 1.

### 2.2. Main Functions of AhR

AhR is a unique and versatile biological sensor of planar chemical compounds of endogenous and exogenous origin [29,30] and is the only member of the PAS family that binds naturally occurring xenobiotics [31]. By functioning as a transcription factor, AhR takes part in many physiological and pathological processes in cells and tissues. 

Traditionally, AhR has been known as a mediator of xenobiotic metabolism ever since AhR was reported to bind to 2,3,7,8-tetrachlorodibenzo-p-dioxin (TCDD). AhR over-activates the transcription of target genes, resulting in a release of many toxic compounds; for example, AhR is an activator of TCDD as a carcinogen [32,33]. For many years, AhR has been a research subject of toxicologists owing to its involvement in the metabolism of environmental pollutants and food contaminants such as polycyclic aromatic hydrocarbons, polychlorinated biphenyls, and dioxins [33,34].

Later, numerous studies have shown that AhR is activated by many natural and synthetic ligands, which may or may not be planar molecules of the polycyclic aromatic hydrocarbon type [35,36]. In this context, AhR acts as a sensor that connects the external environment and internal environment. AhR participates in processes of development, immune defense, and homeostasis, including cell differentiation and physiological processes in stem cells. In these cases, its ligands are various endogenous compounds.

In particular, the endogenous stimulation of AhR determines its main function (in mammals), which is related to the normal development of an organism and its homeostasis under the conditions of chemically diverse and dynamic internal and external environments [37,38,39,40,41]. 

AhR exerts this action by regulating fundamental metabolic processes that modulate cell proliferation, cell cycle, cell differentiation and phenotype formation, and cell adhesion and migration [40,42,43,44,45,46].

The involvement of the AhR in cell cycle regulation confirms its important role in the modulation of cellular homeostasis [33,47,48]. One hypothesis postulates that the endogenous stimulation of AhR triggers the recognition of cellular stress, thereby altering gene expression and causing cell cycle arrest [42,49,50].

In several human cell lines, it has been demonstrated that excessive AhR activation results in cell cycle arrest in the G1 phase; this event makes it impossible for the cell to enter the S phase; this blockade is partly due to a direct interaction of AhR with hypophosphorylated retinoblastoma protein (pRb) [51,52,53,54]. 

The overstimulation of receptor AhR by anthropogenic pollutants leads to a substantial dysregulation of AhR activity and of its downstream cascades [55,56,57]. Apparently, this phenomenon wreaks havoc on the fine regulation of cellular metabolic processes, e.g., owing to the disruption of mitochondrial structure/function and proliferative activity [58,59].

### 2.3. AhR Ligands and Target Genes

AhR is activated by a wide range of ligands (Table 1), which can be categorized into endogenous ligands and exogenous ones [60,61,62]. 

Among the exogenous AhR ligands, halogenated aromatic hydrocarbons are typical, including dioxins (such as TCDD) [63], polychlorinated biphenyls, and polycyclic aromatic hydrocarbons such as benzo[*a*]pyrene (BaP) and 3-methylcholanthrene [33,34]. AhR also binds to a number of drugs such as omeprazole [64] and to compounds present in foods, such as plant polyphenols and flavonoids (e.g., quercetin) [65,66].

Aside from environmental compounds, many small-molecule compounds have been identified that bind to AhR and modulate its activity [33,67].

Growing interest in the physiological functions of AhR has led to the identification of many endogenous ligands of AhR [68]. These include heme metabolites bilirubin and biliverdin [69], tetrapyrroles [70], arachidonic acid metabolites [70,71,72], tryptophan metabolites such as kynurenic acid [73] and kynurenine [68], 6-formylindolo[3,2-*b*]carbazole (FICZ) (which is a photoproduct of the ultraviolet irradiation of L-tryptophan [74]), indolo[3,2-*b*]carbazole [68], and estrogen equilenin [72]. Compounds secreted by bacteria can also be AhR ligands [60,75,76].

AhR target genes code for phase I enzymes that metabolize xenobiotics (e.g., CYP1A1, CYP1A2, and CYP1B1) and phase II enzymes including NQO1, GSTA2, aldehyde dehydrogenase 3A1, UGT1A1, and UGT1A6 [21,67,77,78,79,80]. 

AhR ligands can serve as either agonists or antagonists of the transcription of AhR-controlled genes, depending on various conditions in the cell. In different cell types, there are diverse scenarios of gene activation in response to AhR stimulation. Different AhR ligands can induce dissimilar transcriptome profiles within the same cell type, and the same AhR ligand can give rise to different transcriptome profiles in different cell types [81,82,83].

### 2.4. Pathways of Transcription Regulation by AhR and Crosstalk with Other Signal Transduction Pathways

**Figure 2:**
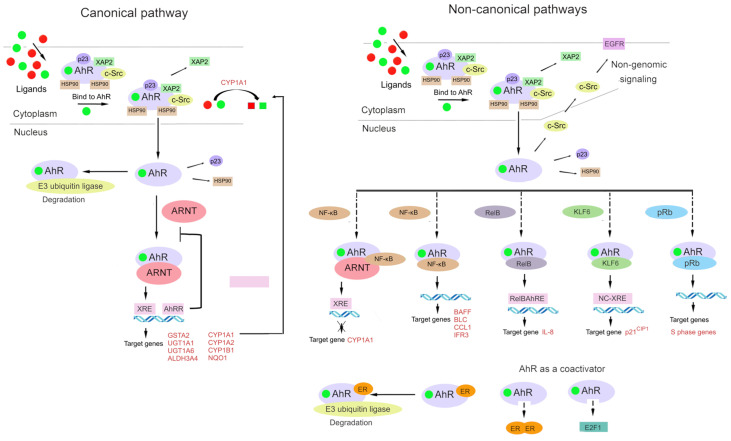
An outline of canonical and non-canonical AhR signaling pathways. Under physiological conditions, AhR is localized to the cytosol and forms a complex with specific proteins, such as hepatitis B virus X-associated protein 2 (XAP-2), heat shock protein 90 (HSP90), cytosolic endoplasmic-reticulum proteins, and protein tyrosine kinase c-Src. After binding to a ligand, AhR changes its conformation and relocates to the nucleus, where it dimerizes with AhR nuclear transporter (ARNT) or other partners such as transcription factor Krüppel-like factor 6 (KLF6) or transcription factors of the nuclear factor kappa B (NF-κB) family (e.g., RelB). Dissociated c-Src interacts with epidermal growth factor receptor (EGFR). AhR signaling is connected with the activity and function of estrogen receptor and E2 promoter-binding factor 1 (E2F1), which is capable of binding to pRB. The AhR–ARNT complex binds to a xenobiotic-responsive element (XRE) and induces the transcription of AhR-controlled genes. Proteins AhR and KLF6 form a heterodimer that recognizes a novel non-consensus XRE (NC-XRE) and initiates the transcription of genes involved in cell cycle regulation. Proteins AhR and RelB (an NF-κB subunit) combine into a heterodimer that recognizes a RelB–XRE complex and induces the transcription of some chemokine genes. AhR and NF-κB form a heterodimer that lead to the inducing of the expression of cytokines and chemokines B-cell-activating factor of the tumor necrosis factor family (BAFF), B-lymphocyte chemoattractant (BLC), CC-chemokine ligand 1 (CCL1), and interferon-responsive factor (IFR3). The AhR/ARNT/NF-κB interaction decreases the expression of CYP1A1. AhR and pRb form a heterodimer that lead to a blocked cell cycle progression by suppressing the expression of S-phase genes. AhR activity is controlled by negative feedback loops, including the metabolism of ligands, the disruption of the AhR/ARNT complex by AhR repressor (AhRR), and proteosomal degradation by the ubiquitin ligase complex. AhR in complex with ER promotes the proteolysis of ER by ubiquitin ligase complex.

The AhR signaling pathway involves both classic (canonical) and non-classic (non-canonical) signal transduction mechanisms (Figure 2) [31,84]. 

The classic (canonical) pathway of xenobiotic metabolism was the first-studied molecular mechanism of AhR action, and adherence to this paradigm has greatly delayed the understanding of the global biological significance of AhR.

Under physiological conditions, AhR is localized to the cytosol and forms a complex with specific chaperone proteins, such as hepatitis B virus X-associated protein 2 (XAP2, also known as AIP or ARA9), p23, and c-Src [24,84,85,86]. Ligand binding results in a conformational change that causes AhR to disassociate from the above complex, and then the ligand–AhR complex is translocated from the cytosol to the nucleus [87,88].

In the classic mechanism of transcriptional regulation, the complex of AhR with its ligand heterodimerizes with ARNT and binds to xenobiotic-responsive elements in DNA upstream of AhR’s inducible target genes. The AhR–ARNT complex initiates the transcription of several genes, including cytochrome P450 family 1 subfamily A member 1 (*CYP1A1*) and subfamily B member 1 (*CYP1B1*), and this action has a wide range of physiological and toxic effects [24,70,89,90,91].

In the non-canonical transcriptional regulatory pathway, the ligand–AhR complex heterodimerizes with partner proteins other than ARNT, for example, Krüppel-like factor 6 (KLF6) and RelB [92,93].

AhR interacts with the signaling pathway of the nuclear factor kappa-light-chain enhancer of activated B cells (NF-κB) [94,95,96]. Through interactions of AhR with RelA or RelB, AhR signaling can promote the activation of NF-κB [97,98,99]. AhR and NF-κB form a heterodimer that lead to the inducing of the expression of cytokines and chemokines B-cell-activating factor of the tumor necrosis factor family (BAFF), B-lymphocyte chemoattractant (BLC), CC-chemokine ligand 1 (CCL1), and interferon-responsive factor (IFR3) [100,101]. 

Additionally, AhR can interact with other signal transduction pathways. There seems to be bidirectional crosstalk between AhR and nuclear factor erythroid 2-related factor 2 (Nrf2) [102,103]. The *Nrf2* gene promoter contains at least one functional xenobiotic-responsive element [104], whereas the *AhR* gene promoter has several antioxidant response elements (AREs) [103]. The crosstalk of the AhR and Nrf2 pathways is discussed in detail below.

AhR signaling is linked with estrogen receptor activity and function [21,67], for which a ligand–AhR complex can serve as a coactivator [92,105]. Additionally, a ligand–AhR complex can function as a coactivator of E2 promoter-binding factor 1 (E2F1): a transcription factor that is crucial for the cell cycle transition from the G1 phase to the S phase [92,106]. The binding of AhR to the hypophosphorylated “active” form of the retinoblastoma tumor suppressor protein (pRb) leads to cell growth arrest in the G1/S phase of the cell cycle [107]. It is reported that two mechanisms contribute to this effect. In the first one, pRb acts as a transcriptional coactivator of classic induction of CYP1A1 by dioxin-like ligands. In the second mechanism, AhR is a component of a repressor complex along with pRb, E2F, and partner protein E2F DP [89,108,109]. 

Aside from genomic signaling via target genes [33,67,84,110,111], AhR participates in nongenomic signaling [46]. For example, upon the binding of a ligand to a cytosolic complex of AhR with chaperones, kinase c-Src can be released, which relocates to the plasma membrane, thereby activating EGF signaling [66,112].

It has been revealed that certain compounds can directly induce the expression of AhR target gene *CYP1A1*, suggesting that AhR activation can occur in the absence of direct ligand binding [113]. Indeed, nongenomic effects of AhR have been documented, especially in the context of the induction of inflammatory processes. For instance, TCDD has been reported to increase intracellular calcium concentration, thereby initiating a cascade of reactions ultimately causing cyclooxygenase (COX) 2 activation and an accumulation of inflammatory mediators such as prostaglandins [114,115]. Moreover, AhR is reported to mediate the toxic cellular effects of TCDD through pro-oxidant mechanisms [34]. 

## 3. AhR Regulates Enzyme Systems Generating Reactive Oxygen Species

AhR is reported to be responsible for the toxic cellular effects of TCDD via pro-oxidant mechanisms [34,116]. There is convincing evidence that the activation of AhR-dependent detoxification of such environmental stressors as TCDD, polycyclic aromatic hydrocarbons, polychlorinated biphenyls, and effects of ultraviolet radiation gives rise to oxidative stress and to the production of reactive oxygen species, thus inducing oxidative damage to DNA, lipids, and other cellular macromolecules [117,118,119,120]. Several enzyme systems, including CYP1A, NOX, COX, and possibly aldo–keto reductase (AKR) 1, are regulated through the AhR signaling pathway in terms of their ability to generate reactive oxygen species in various cell types and tissues [121,122,123,124].

### 3.1. CYP1A

The production of reactive oxygen species by cytochromes P450 is associated with a catalytic circle of enzymes, to be precise, with a phenomenon called “uncoupling” [125,126,127]. In the presence of NADPH, CYP monooxygenases reduce molecular oxygen, where one oxygen atom is attached to the substrate, and the second one is reduced to form a water molecule. Stoichiometric analysis of this reaction shows that most CYP enzymes consume more oxygen than necessary to mono-oxygenize their substrate, and hydrogen peroxide can be a byproduct of this reaction [128,129]. 

When compounds with a stable structure induce the formation of a complex of CYP with oxygen, the absence of an electron acceptor can cause auto-oxidation of CYP and a subsequent release of a superoxide anion radical which dismutates, thereby yielding hydrogen peroxide too. As a result of Fe^2+^-catalyzed Haber–Weiss and Fenton reactions, both superoxide anions and hydrogen peroxide can be converted into highly reactive hydroxyl radicals [125]. 

The interaction of TCDD with AhR enhances the expression of such cytochrome P450 family members as CYP1A1, CYP1A2, and CYP1B1. Due to the stable structure of TCDD, these enzymes are unable to metabolize it efficiently. In addition, the formation of reactive oxygen species is caused by excessive CYP1A1 activity resulting from the binding of TCDD to AhR [21,130]. For instance, the AhR-dependent induction of CYP1A is the main source of reactive oxygen species in hepatocytes incubated with TCDD [119]. Similarly, exposure to polycyclic aromatic hydrocarbons such as BaP causes CYP1A1 the overexpression and production of reactive oxygen species [131].

The overproduction of reactive oxygen species under the influence of CYP1A1 may indirectly affect cell metabolism, owing to the direct activation of several signaling pathways. Moreover, the interaction of reactive oxygen species with various biomolecules, such as NF-κB or oncoprotein c-Jun or Rb, can affect the cell cycle [132,133].

### 3.2. NADPH Oxidases

The metabolic activation of polycyclic aromatic hydrocarbons involves NADPH oxidase in addition to CYP1 isoforms [134]. There is evidence that polycyclic aromatic hydrocarbons can stimulate the production of reactive oxygen species via NADPH oxidases, in particular NOX2 and NOX4 [135,136,137,138,139,140].

These membrane-bound enzyme complexes are detectable in the plasma membrane of various cell types, such as phagocytes and endothelial and epithelial cells [141]. In the inactive state, NADPH oxidase subunits—three cytoplasmic (Rac1, p47^phox^, and p67^phox^) and two intramembrane ones (p22^phox^ and gp91^phox^)—are not assembled [141]. After activation by cytokines, by opsonized bacteria, by bacterial lipopolysaccharides, or by other stimuli, the complex assembles and the catalytic subunit, i.e., the heterodimeric flavocytochrome composed of gp91^phox^ and p22^phox^, and transfers one electron from NADPH to molecular oxygen, thus yielding superoxide anions, which are next dismuted into hydrogen peroxide [141,142,143].

Additional proteins, such as p40^phox^ (one of NADPH oxidase subunits), play an important part in the regulation of NADPH oxidase activity and in the subsequent production of reactive oxygen species [140,141].

According to the literature, there are several mechanisms of NADPH oxidase activation through the AhR signaling pathway. For example, the 0NOX2-mediated formation of reactive oxygen species in epidermal keratinocytes under the action of a polycyclic aromatic hydrocarbon is mediated in an AhR-dependent way by the stimulation of the phosphorylation of p47^phox^ (neutrophil cytosolic factor 1), which is necessary for the assembly of the NOX2 complex on the plasma membrane [137]. In a study on the liver of C57BL/6J mice treated with 3-MX, induction of the NADPH oxidase subunit p40phox was observed, which was not the case in the liver tissue of mice with a conditional AhR b knockout in the liver [140]. In an analysis of Hepa1c1c7 cells, a functional xenobiotic-responsive element was detected in the promoter of the murine p40phox gene [140].

Another mode of NADPH oxidase activation in human and rat macrophages involves the increased transcription of p47phox because of the direct binding to XRE in the promoter region of this gene after treatment with BaP. In addition, BaP promotes the translocation of the p47^phox^ protein to the macrophage plasma membrane and strengthens the production of superoxide anion under the influence of phorbol myristate acetate [136].

Reactive oxygen species that are generated in epidermal keratinocytes during exposure to a polycyclic aromatic hydrocarbon initiate mitogen-activated protein kinase (MAPK) signaling, which drives the activation of transcription factors AP-1 and NF-κB and the subsequent initiation of proinflammatory processes [137].

It has also been shown that AhR ligands, such as TCDD and dioxin-like planar polychlorinated biphenyls, or endogenous substances (e.g., indoxyl sulfate or arachidonic acid) activate NADPH oxidase and thus stimulate the production of reactive oxygen species, thereby leading to damage to vascular endothelial cells [144,145]. During the incubation of human umbilical vein endothelial cells with the endogenous AhR ligand indoxyl sulfate, the production of reactive oxygen species increases through the overexpression of NOX4, thus damaging these cells [146].

NOX4 activation by thiol-reactive agents such as cadmium, arsenic, nickel, and mercury interferes with AhR signaling [147]. For example, the treatment of human HaCaT keratinocytes with arsenic results in NOX4-dependent oxidative stress. The subsequent inhibition of the catalytic activity of CYP1A1 by reactive oxygen species induces an accumulation of the endogenous AhR ligand 6-formylindolo[3,2-b]carbazole and to an AhR-dependent increase in CYP1A1 transcription [130,147]. The same effect is observed in arsenic-treated murine cells [148,149]. 

The influence of reactive oxygen species on the metabolic degradation of AhR ligands may also explain the high transcriptional activity of AhR that is observed in glutathione-depleted normal and malignant breast cells [150].

### 3.3. Cyclooxygenase

In the biosynthesis of prostaglandin E_2_, cyclooxygenase is a key rate-limiting enzyme that catalyzes the conversion of arachidonic acid to prostaglandins [151,152]. Furthermore, there is an alternative enzyme for chemical oxidation: prostaglandin endoperoxide synthase 2, also known as COX2. The latter is an example of an alternative enzyme for xenobiotic metabolism in extrahepatic tissues [153,154]. 

The activation of AhR by TCDD has been found to induce the expression and activity of COX2 [155,156]. Unlike COX1 expression, the expression of COX2 can be induced by various stimuli, such as growth factors and cytokines [157]. The upregulation of COX2 has been implicated in chronic inflammation and carcinogenesis [158,159,160].

COX2 converts arachidonic acid to prostaglandin (PG) G_2_, which undergoes peroxidation to PGH_2_. In this two-step enzymatic process that generates reactive oxygen species, the cyclooxygenase is the rate-limiting enzyme for the formation of prostaglandins [152,161].

Although TCDD and other AhR ligands drive CYP1A1 and CYP1A2 expression via the canonical AhR–ARNT pathway, TCDD-induced expression of COX2 involves non-canonical AhR signaling pathways such as c-Src activation and the subsequent binding of CCAAT/enhancer-binding protein β [162] and MAPK signal transduction [137,163]. 

The overexpression of COX2 may enhance the production of reactive oxygen species. Elevated levels of COX2 and reactive oxygen species can cause vasoconstriction and renal endothelial dysfunction [164]. In another study, it was hypothesized that lipopolysaccharide inhibits the endothelium-dependent vasodilatory response in middle cerebral arteries of normotensive rats [165]. The effect of lipopolysaccharide in that work was mediated by a release of the superoxide anion that was generated, at least in part, via lipopolysaccharide-induced expression of COX2.

### 3.4. Aldo–Keto Reductases

The metabolic activation of polycyclic aromatic hydrocarbons involves aldo–keto reductases in addition to CYP1 isoforms, and aldo–keto reductases participate in the formation of reactive oxygen species. These enzymes are cytosolic NADPH-dependent oxidoreductases that convert carbonyl groups to primary and secondary alcohols [166,167].

Aldo–keto reductases, in particular human AKR1A1 and AKR1C1–AKR1C4, can oxidize *trans*-dihydrodiols (which are intermediates of CYP1-mediated oxidation of polycyclic aromatic hydrocarbons) to the corresponding catechols [3,168]. For example, BaP is oxidized by CYP1A1 to BaP-7,8-epoxide [169]. In a dihydroxylation reaction, microsomal epoxide hydrolase 1 transforms this epoxide into BaP-7,8-*trans*-dihydrodiol, which is detoxified by phase II enzymes, re-oxidized by CYP1 isoforms, or converted by aldo–keto reductases into BaP-7,8-catechol [166,169]. In the presence of oxygen, BaP-7,8-catechin undergoes one-electron oxidation, giving rise to the o-semiquinone anion radical and resulting in a release of hydrogen peroxide [170]. If the o-semiquinone anion radical is not detoxified by catechol-O-methyltransferases or phase II conjugating enzymes, another one-electron oxidation gives BaP-7,8-dione (o-quinone) and superoxide anion radicals [170]. BaP-7,8-dione is highly reactive, and either forms DNA adducts [171] or undergoes a redox cycle, i.e., is reduced back to BaP-7,8-catechin, in the presence of NADPH [172].

Consequently, the aldo–keto reductase-mediated metabolism of polycyclic aromatic hydrocarbons contributes to oxidative damage and their genotoxicity.

The role of AhR in the regulation of *AKR1* gene expression is not yet clear. High expression of AKR1C enzymes is observed in BaP-exposed cell lines, including human hepatoma, colon carcinoma, and breast cancer cells [122,123,124]. Moreover, an AhR knockdown in breast cancer MDA-MB-231 cells drastically lowers both basal and 3-methylcholanthrene-induced AKR1C3 expression [124]. On the other hand, the AhR ligand prototype TCDD is known to be ineffective in terms of AKR1C induction, and promoter sequences of the gene encoding human AKR1C do not contain sensitive xenobiotic-responsive elements [122,123,124]. suggesting that the regulation of these enzymes is not mediated by the canonical AhR–ARNT signaling pathway.

## 4. Participation of AhR in Antioxidant Defense

Aside from the AhR-dependent production of intracellular reactive oxygen species, the AhR signaling pathway modulates the expression of genes of the antioxidant system and thereby regulates cell functions that ensure protection from oxidative stress. Numerous studies indicate that the protective action of antioxidants against oxidative stress is mediated by AhR through a response to such AhR ligands as flavonoids, phytochemicals, and azoles [173,174,175,176,177,178,179,180]. 

When this type of ligand binds to AhR, the production of reactive oxygen species does not occur because of the induction of the nuclear translocation of AhR; instead, Nrf2 is activated. Nrf2 is a key biomolecule that provides cell protection against the oxidative damage caused by reactive oxygen species: Nrf2 is a transcription factor that regulates the genes encoding enzymes of the antioxidant system [102,181]. 

### 4.1. Nrf2 Expression, Functions and Signaling

In a normal physiological state, Nrf2 is located in the cytoplasm and binds to Kelch-like ECH-associated protein 1 (Keap1) [182,183]. In response to stress signals, Keap1 is inactivated, resulting in Nrf2 stabilization. Nrf2 is translocated to the nucleus where it binds to members of the Maf protein family (e.g., MafK, MafF, and MafG). In a sequence-specific manner, the Nrf2–sMaf complex binds to an ARE, 5′-TGACXXXGC-3′, in a promoter region and activates the transcription of target genes of Nrf2 [19,181,184,185,186].

Nrf2 regulates the transcription of genes encoding components of the antioxidant systems based on glutathione and thioredoxin as well as genes coding for enzymes involved in the phase II detoxification of exogenous and endogenous compounds or in NADPH regeneration and heme metabolism (heme oxygenase 1): GPX4, superoxide dismutase, sulfiredoxin, paraoxonases, NQO1, GSTP1, GSTA1/2, UGT1A6, and various other enzymes that remain to be identified [187,188,189,190,191,192].

In addition to ensuring redox homeostasis, Nrf2 functions encompass multiple cellular processes, including the regulation of cell survival, metabolic and protein homeostasis, inflammation, and cell proliferation and differentiation [193,194,195,196,197]. 

Nrf2 is at the center of a complicated regulatory network. Its activity is modulated at several levels, including transcriptional regulation (by NF-κB, AhR, ATF4, and other transcription factors and cofactors), post-transcriptional regulation (by microRNA, RBPs, or alternative splicing), post-translational regulation (by ERK, JNK, PKC, CK2, PERK, GSK3, or p38), and the regulation of Nrf2 protein stability (by KEAP1, βTrCP, HRD1, WDR23, or CRIF1) [181,198]. 

### 4.2. Participation of AhR in Mechanisms of Nrf2 Activation

**Figure 3:**
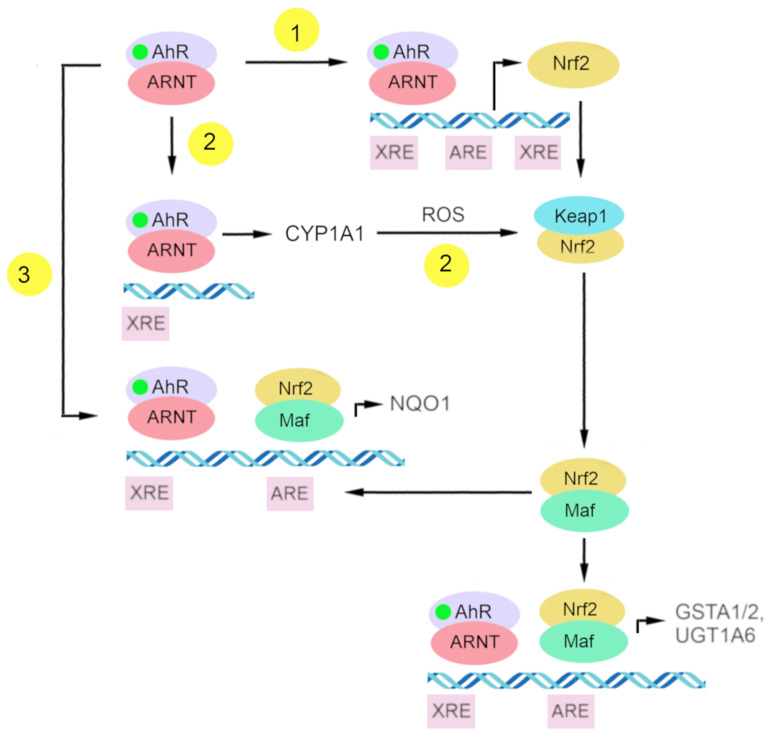
The scheme of putative connections between gene batteries of AhR and Nrf2. (1) *Nrf2* is a target gene of AhR; (2) indirect activation of Nrf2 by CYP1A1-generated reactive oxygen species (ROS); and (3) direct interaction of complexes AhR–XRE and Nrf2–ARE in a regulatory region of the *NQO1* gene.

At present, there is some understanding of the mechanisms underlying Nrf2 activation by AhR. (Figure 3) One of them is the transcriptional activation of *Nrf*2 as a target gene of AhR, and the other is the indirect activation of Nrf2 via CYP1A1-generated reactive oxygen species [199]. 

#### 4.2.1. Nrf2 as a Target Gene of *AhR*

AhR is one of the transcription factors regulating *Nrf2* [199,200]. Research on the *Nrf2* promoter indicates that *Nrf2* is a target gene of AhR. That *Nrf2* gene transcription is directly modulated by AhR activation has been demonstrated by DNA sequence analyses of the mouse *Nrf2* promoter; this work revealed one xenobiotic-responsive element-like element (XREL1) located at position –712 and two additional xenobiotic-responsive element-like elements located at positions +755 (XREL2) and +850 (XREL3). In those studies, functional analysis by a luciferase assay revealed that XREL1, XREL2, and XREL3 are all inducible by TCDD treatment, with XREL2 being the most potent [104,200]. 

There is also confirmation of the functionality of these xenobiotic-responsive element-like elements, and a direct binding of AhR to the *Nrf2* promoter has been proven [200]. It has been reported that *Nrf2* expression is at least partly regulated by AhR inducers through the activation of multiple xenobiotic-responsive elements in the *Nrf2* promoter. This molecular event represents a direct connection between AhR and Nrf2 and places the Nrf2–ARE pathway downstream of AhR–XRE activation in certain scenarios [104].

There is fine-tuned crosstalk between AhR and Nrf2, which mutually enhance or weaken their activation states. The antioxidant response resulting from AhR activation and mediated by Nrf2 depends on the type of AhR ligand. Such AhR ligands as dioxins, BaP, and other polycyclic aromatic hydrocarbons bind to AhR with high affinity and induce extremely high *CYP1A1* expression along with reactive oxygen species production [131,201]. Although Nrf2 is also activated in this case [102], the oxidative stress suppresses antioxidant defense [131,177]. 

Other AhR ligands, such as phytochemicals and flavonoids, bind to AhR less strongly [202,203,204], and among antioxidant phytochemicals, there are those that activate the AhR signaling pathway without the production of reactive oxygen species. These include ketoconazole and cynaropicrin [173,174]. 

Antioxidant phytochemicals capable of modulating Nrf2, AhR, and CYP1A1 have been described. By means of epidermal keratinocytes, as an example, it has been revealed that phytochemicals exerting their antioxidant actions through AhR and Nrf2 signaling can be categorized into three groups depending on their ability to increase and decrease AhR and CYP1A1 activities [10]. Group 1 contains Nrf2 agonists with AhR-agonistic activity. This group includes soybean tar, Opuntia Ficus-Indica extract, *Houttuynia cordata* extract, Bidens pilosa extract, and cynaropicrin. Group 2 contains Nrf2 agonists with AhR-antagonistic activity. This group includes cinnamaldehyde and epigallocatechin gallate. Group 3 contains Nrf2 agonists with CYP1A1-inhibitory activity. This group includes Z-ligustilide, quercetin, kaempferol, pterostilbene, and resveratrol.

Nonetheless, the ability of phytochemicals to regulate Nrf2, AhR, and CYP1A1 functions may depend on the cell type. The exact mechanisms by which these compounds influence the AhR and Nrf2 pathways differently remain unknown [10,100,199,205]. 

#### 4.2.2. Indirect Activation of Nrf2 via CYP1A1-Generated Reactive Oxygen Species 

Another mechanism of Nrf2 activation by AhR is the indirect activation of Nrf2 via CYP1A1-generated reactive oxygen species. The upregulation of intracellular reactive oxygen species can lead to the oxidation of Keap1 and a release of Nrf2 from its complex [199,206].

#### 4.2.3. Direct Crosstalk between AhR–XRE and Nrf2–ARE Signaling Pathways 

AhR and Nrf2 signaling pathways coordinate the expression regulation of genes of phase II xenobiotic metabolism, e.g., *GSTA2*, *UGT1A6*, and *NQO1* [191,199,200,207,208,209]. The mechanism of direct crosstalk between the AhR–XRE and Nrf2–ARE signaling cascades has been described for the NQO1 enzyme and involves the close proximity of a xenobiotic-responsive element and ARE in the regulatory region of the *NQO1* gene [199,210]. 

## 5. AhR in the Pathogenesis of Diseases Related to Oxidative Stress 

Although initial studies on AhR were focused on its function as a signaling molecule of a chemical sensor responsive to environmental pollutants, lately, the range of subject areas has widened significantly. Our understanding has expanded regarding the role of the AhR signaling pathway in the regulation of a variety of physiological and pathological phenomena. AhR’s functions cover many cellular processes, including the regulation of cell survival, metabolic and protein homeostasis, inflammation, cell proliferation and differentiation, apoptosis, and cellular adhesion and migration. Reactive oxygen species-induced activation of transcription factors and proinflammatory genes increases inflammation. Accordingly, research on various diseases in which AhR induces an oxidative stress response—by switching on inflammation and antioxidant, prooxidant, and cytochrome P450 enzymes—is now within the scope of the interest of investigators.

It is known that oxidative stress causes inflammation and toxicity, and these problems can lead to such pathologies as cardiovascular, liver, kidney, lung, brain, eye, skin, and joint diseases, as well as aging and cancer [7,211,212,213,214,215]. In recent decades, AhR has been increasingly recognized as an important modulator of disease because of AhR’s role in the regulation of the redox system and of immune and inflammatory responses [120,216].

Below are examples of AhR involvement in the pathogenesis of many human diseases associated with oxidative stress.

### 5.1. Neurological Diseases

Oxidative stress has been studied in neurological diseases, including Alzheimer’s disease, Parkinson’s disease, multiple sclerosis, and amyotrophic lateral sclerosis, and in some psychiatric disorders such as depression [213,215,217,218,219,220]. It is now proven that AhR is involved in the initiation of oxidative stress in the brain because AhR activation by some ligands shifts the cellular redox balance toward oxidative stress [120,221,222]. In most areas of the brain and in the pituitary gland, AhR activation induces CYP1A1 and CYP1B1 expression [223]. This event can result in the mitochondrial production of reactive oxygen species [124,224] and in the higher production of reactive oxygen species through the activation of the arachidonic acid pathway by CYP enzymes and other intracellular signaling processes [70,225].

AhR plays an important part in the initiation of benign and malignant brain tumors [226,227]. In glioblastoma cells, neuroactive hormone dopamine has been identified as an inducer of enzymes CYP1A1, CYP1B1, and UGT1A1 [228].

It has been proven that AhR signaling pathways (especially after activation by such endogenous AhR ligands as tryptophan metabolites) are implicated in neurodegenerative diseases, in particular in well-known age-related brain diseases (Parkinson’s disease, Alzheimer’s disease, multiple sclerosis, and amyotrophic lateral sclerosis) and other diseases of the central nervous system [229,230,231,232,233]. The hypothesis that AhR is involved in the neurodegenerative processes in Parkinson’s disease and Alzheimer’s disease derives from both human and in vitro studies. An important question addressed in these studies is the contribution of environmental factors to the risk of neurodegenerative diseases [67,233,234]. 

Parkinson’s disease is an extrapyramidal disorder characterized by decreased motor function due to the loss of dopaminergic neurons. Toxic exogenous ligands such as TCDD enhance the degeneration of dopaminergic neurons in the midbrain owing to enhanced oxidative stress, thereby inducing experimental Parkinson’s disease; in contrast, several phytochemicals such as a flavonoid called tangeretin as well as natural compounds from the plant *Withaferin sominifera* act via AhR to protect against Parkinson’s symptoms in several models of this disease [235,236]. An experimental murine model of Parkinson’s disease has revealed that AhR activation by BaP may have a protective effect against this pathology [237]. It should be noted that AhR is activated by carbidopa, which is used to treat Parkinson’s disease [238]. 

AhR is associated with Alzheimer’s disease too, which is a neurodegenerative illness featuring aggregation of β-amyloid plaques, which cause neuroinflammation and promote neuronal loss [239]. In a mouse model of Alzheimer’s disease, AhR activation alleviates cognitive deficits through the upregulation of neprilysin: the main endogenous enzyme of β-amyloid catabolism [239]. Those authors showed that neprilysin expression and enzymatic activity are higher when AhR is activated by endogenous ligands L-kynurenine or 6-formylindolo[3,2-*b*]carbazole or by exogenous ligands diosmin or indole-3-carbinol; they also found that AhR is a direct transcription factor of the neprilysin gene [239]. Substantial amounts of AhR and indolamine-2,3-dioxygenase 1 (IDO1) are detectable in glial cells in postmortem brain samples from Alzheimer’s patients and in postmortem hippocampus and serum samples from such patients; these amounts are elevated as compared with young people and elderly patients without dementia [230]. 

It has been established that β-amyloid neurotoxicity depends on AhR activation through the IDO1–kynurenine–AhR cascade, where—via the increased activity of IDO1 (an enzyme responsible for tryptophan degradation)—the production of tryptophan metabolites is accelerated, which can act as AhR ligands and may be neurotoxic [240]. On the contrary, inhibitors of IDO1 attenuate the neurotoxic activity of AhR. Because of the evidence of the neuroprotective effects of AhR against neurodegenerative diseases of the brain, research on non-toxic AhR agonists will be necessary and may help to alleviate the symptoms as a new therapeutic strategy against these diseases.

In multiple sclerosis and amyotrophic lateral sclerosis, the enhancement of inflammatory and neurodegenerative processes in the central nervous system is caused by environmental factors, among other reasons. In particular, the risk of multiple sclerosis correlates with smoking, which drives *AhR* gene demethylation and inhibits AhR signaling pathways, followed by the enhancement of inflammatory processes in the central nervous system in multiple sclerosis [241,242,243]. Patients with multiple sclerosis have lower levels of circulating AhR than healthy controls do, suggesting that AhR is involved in the pathogenesis of this disorder [244]. AhR may limit the central nervous system-inflammation characteristic of multiple sclerosis by suppressing astrocyte activation [245,246]. In multiple sclerosis, the gut microbiome is altered, which represents an interesting opportunity for investigating the role of the kynurenine–AhR pathway in this pathology [247]. In an animal model of multiple sclerosis (autoimmune encephalomyelitis), it has been shown that AhR may be a therapeutic target in multiple sclerosis too: a knockdown of AhR worsens disease signs, whereas AhR activation by such AhR agonists as TCDD, indole-3-carbinol, or diindolylmethane slows the progression of experimental allergic encephalomyelitis, owing to the overexpression of Forkhead Box P3, greater numbers of anti-inflammatory regulatory T cells, and the attenuation of proinflammatory expansion of T helper 17 (Th17) cells [246,248,249]. 

6-Formylindolo[3,2-*b*]carbazole, another AhR agonist, also alleviates disease progression when systemically administered in mouse models [250]. On the other hand, topical administration of 6-formylindolo[3,2-*b*]carbazole promotes Th17 cell expansion, thus worsening disease signs [249]. Furthermore, in an animal model of multiple sclerosis (autoimmune encephalomyelitis), treatment with laquinimod, which crosses the blood–brain barrier, reduces astrogliosis and prevents the production of downstream proinflammatory cytokines in an AhR-dependent manner [251]. These results suggest that laquinimod is a first-in-class drug that targets AhR for the treatment of multiple sclerosis and other neurodegenerative diseases.

In amyotrophic lateral sclerosis, TDP-43 has been identified as the major pathological protein [252]. Drugs targeting this protein have become a therapeutic approach to this disease. One work on a cell line (induced pluripotent stem cells differentiated into motor neurons) on a mouse brain and on human neuronal cell lines (BE-M17 cells) has revealed that AhR activation by an exogenous ligand (TCDD) or an endogenous ligand (6-formylindolo[3,2-*b*]carbazole) raises the TDP-43 protein level in human neuronal cell lines and motor neurons [243]. The observed effects were abrogated by AhR antagonists, implying that exposure to the environmental toxic substances that activate AhR may be a risk factor for the onset or progression of amyotrophic lateral sclerosis [243].

Available data suggest that AhR activation may have a bidirectional effect on diseases of the brain. At the same time, only the IDO1-kynurenine-AhR cascade has been clearly shown to be involved in the development of Alzheimer’s disease. AhR function may depend on additional stress events, or cell types. Effects on the AhR signaling pathway may depend on interaction with various coactivators or ligand-selective binding of the AhR complex to nontraditional sequences of XRE [226,253]. Apparently, in different cases, AhR antagonists or agonists can promote or hinder the development of neurodegenerative disorders, as well as exert either pro- or antitumor influence. 

### 5.2. Ocular Diseases 

Oxidative stress participates in age-related macular degeneration and cataracts by altering various types of eye cells photochemically or nonphotochemically [254]. Under the action of free radicals, crystalline proteins in the lens can become crosslinked and aggregate, causing the formation of cataracts [255]. In the retina, prolonged exposure to radiation can inhibit mitosis in the retinal pigment epithelium and choroid, may damage outer segments of photoreceptors, and can be implicated in lipid peroxidation [256].

Because environmental factors and metabolites have been shown to affect the central nervous system, researchers have demonstrated a role of AhR in some diseases of the central nervous system and a functional contribution of AhR to the regulation of the behavior of astrocytes, other microglial cells, and neurons; given that the retina is an extension of the brain, a growing area of research deals with the involvement of AhR in ocular diseases [233].

The importance of AhR and AhR signaling in eye development, toxicology, and diseases is currently being uncovered. AhR is expressed in many ocular tissues, including the retina, choroid, cornea, and orbit. A considerable role of AhR in age-related macular degeneration, glaucoma, and other eye diseases has been identified [257].

Glaucoma is caused by an increase in intraocular pressure, which damages the optic nerve and induces vision loss and blindness. *CYP1B1*, an AhR-inducible gene, is associated with various types of glaucoma, including two main ones: primary open-angle glaucoma and primary congenital glaucoma [258,259]. Mutations correlating with various types of glaucoma have been identified in *CYP1B1* and cause a low CYP1B1 enzymatic activity or its absence [258,260].

Additionally, TCDD can induce CYP1B1 expression in non-pigmented human ciliary epithelial cells that constitute the ciliary body, whose main function is the production of aqueous humor in the eye [261]. CYP1B1 takes part in the metabolism of steroids, retinol, retinal, arachidonate, and melatonin. Consequently, CYP1B1 expression, which is elevated during AhR activation, alters the biosynthesis of critical metabolites as well as metabolic pathways that may lead to the initiation and/or progression of glaucoma [258].

Age-related macular degeneration is a complex multifactorial disease of the elderly and has unclear pathogenesis. In age-related macular degeneration, one of the destructive processes is oxidative stress, which gives an imbalance among the processes responsible for the production and detoxification of reactive oxygen species. In the dry type of age-related macular degeneration, there is a thinning and degradation of choroid capillaries, expanding atrophy of the outer retinal part, and irreversible damage to photoreceptors. In wet age-related macular degeneration, the presence of proinflammatory cytokines, in particular vascular endothelial growth factor (VEGF), promotes angiogenesis and vascular permeability.

During an investigation into the AhR function in retinal homeostasis, a loss of AhR signaling was found to increase retinal susceptibility to environmental stressors such as intense light [262]. In the retina of *Ahr^−/^^−^* mice, there is a subretinal microglia accumulation concurrent with changes in autofluorescence, degeneration of the retinal pigment epithelium, and immune activation [262]. That study implies that AhR plays a protective part in the retina as a sensor of environmental stress, whereas altered AhR function may contribute to the progression of age-related macular degeneration in humans.

The effects of AhR have been analyzed in retinal pigment epithelial and choroid cell lines using *AhR* small interfering RNA [263]. That paper indicates that the expression of the *VEGFA* gene and of a proinflammatory chemokine (CC motif ligand 2; CCL2) goes up after AhR depletion in a human retinal pigment epithelial cell line, ARPE-19 [263]. Additionally, in that study, AhR depletion increased collagen IV synthesis and secretion in ARPE-19 cells and choroidal RF/6A cells. The depletion of AhR in RF/6A cells also raised the expression of the macrophage chemotactic factor gene, of secreted phosphoprotein 1 (SPP1), and of transforming growth factor (TGF)-β, while reducing the expression of antiangiogenic factor *SERPINF1* [263].

Mice treated with TCDD show elevated VEGFA levels and choroidal vascularization: a hallmark of age-related macular degeneration [264]. These findings suggest that either a loss of AhR expression or AhR activation by TCDD (and/or by other toxic substances in cigarette smoke) promotes angiogenesis, inflammation, and alterations in the extracellular matrix, all of which are seen in the wet type of age-related macular degeneration. 

In an experimental model of age-related macular degeneration (OXYS rats with signs of age-related macular-degeneration-like retinopathy of varying severity), it has been found that an imbalance between the prooxidant (AhR-dependent) system and antioxidant (Nrf2-dependent) system may be key to the pathogenesis of age-related macular degeneration and its initiation and/or progression [265]. Moreover, mitochondria-targeted antioxidant SkQ1 has been shown to ameliorate clinical signs of retinopathy in OXYS rats (manifesting signs of age-related macular-degeneration-like retinopathy) by influencing the transcriptional activity of AhR and Nrf2 and mRNA expression levels of *CYP1A2* and *CYP1B1* in the retina of OXYS and Wistar rats. These data are evidence that the enzymes CYP1A2 and CYP1B1 are implicated in the pathogenesis of age-related macular-degeneration-like retinopathy in OXYS rats and are possible therapeutic targets of SkQ1 [266].

Investigation into the impact of melatonin on mRNA expression of genes of AhR and Nrf2 signaling pathways in OXYS rats has shown that melatonin reduces the mRNA level of AhR-dependent genes of cytochromes CYP1A2 and CYP1B1 in the retina but does not affect mRNA expression of Nrf2-dependent genes in OXYS rats. This means that in age-related macular degeneration, the efficacy of melatonin is attributable to its ability to modulate the expression of AhR signaling pathway genes [267].

The discovery of a new synthetic AhR ligand (2,2′-aminophenylindole) should also be mentioned regarding the development of therapeutic strategies promoting cell homeostasis during the degeneration of the retinal pigment epithelium layer [268]. This compound protects retinal pigment epithelium cells from lipid peroxidation cytotoxicity in vitro and the retina from light-induced damage in vivo. In addition, metabolic characterization of this agent by liquid chromatography coupled with mass spectrometry suggests that 2,2′-aminophenylindole alters lipid metabolism in retinal pigment epithelium cells, thereby increasing the intracellular concentration of palmitoleic acid. The latter, as a downstream effector of 2,2′-aminophenylindole-mediated activation of AhR, has also been reported to protect cells of the retinal pigment epithelium from 4HNE-mediated stress and light-induced retinal degeneration in mice [268].

The synthetic AhR agonist 2,2′-aminophenylindole also regulates microglial homeostasis, thus making AhR a potential target for immunomodulatory and antioxidant therapies. This notion has been illustrated in a study on the anti-inflammatory and antioxidant effects of 2,2′-aminophenylindole on microglial reactivity; the results showed that 2,2′-aminophenylindole strongly represses the expression of proinflammatory genes and induces antioxidant genes in activated human and murine microglial cells, in lipopolysaccharide-stimulated explants of the retina, and in stressed human ARPE-19 cells [269].

### 5.3. Pulmonary Diseases

There is now firm proof that inflammatory lung disorders such as asthma and chronic obstructive pulmonary disease are characterized by systemic and localized chronic inflammation and oxidative stress [270,271,272,273]. Oxidants may contribute to inflammation by activating various kinases and redox transcription factors such as NF-κB and AP-1 [272,273].

The production of an inflammatory mediator called bronchial mucin-containing mucus is usually mediated by a release of cytokines or lipid mediators or by an increase in reactive oxygen species levels [274,275,276,277]. AhR is expressed in many lung cells, including macrophages, club cells, type II alveolar cells, and endothelial cells, and plays an important part in lung function modulation [278]. AhR serves as a regulator of mucosal-barrier function and may influence an immune response in the lungs through changes in gene expression, in intercellular adhesion, in mucin production, and in cytokine expression. AhR is expressed in cells of innate immune responses and in cells crucial for adaptive immunity [278]. 

By means of gene-deficient mice and the administration of AhR agonists and antagonists, numerous researchers have demonstrated that AhR modulates an immune response in various respiratory diseases and that lungs are sensitive to AhR ligands [279,280,281]. Allergic and inflammatory diseases such as bronchitis, asthma, and chronic obstructive pulmonary disease were recently linked with exposure to environmental toxic compounds. AhR mediates the effects of these substances through the arachidonic acid pathway, cell differentiation, intercellular adhesion interactions, cytokine expression, and mucin production. Human bronchial epithelial cells are reported to express AhR, and AhR activation induces mucin production via reactive oxygen species [277,279,282,283,284].

Chronic obstructive pulmonary disease develops as a result of exposure to risk factors that cause oxidative stress, inflammation, and the aberrant proliferation, death, and aging of lung cells, with the consequent destruction of parenchymal tissue [285,286]. AhR has a ligand-specific impact on the lungs and can either aggravate or ameliorate chronic obstructive pulmonary disease. For example, the toxic effects of dioxins and polycyclic aromatic hydrocarbons from tobacco smoke and from particulate matter on the lungs are mediated by AhR signal transduction. These ligands induce inflammation, increase the expression of mucin 5AC and matrix metalloproteinases (MMPs), and damage ciliated cells, club cells, and alveolar macrophages, thereby contributing to the pathogenesis of chronic obstructive pulmonary disease [287,288,289]. 

The exact molecular mechanisms behind the pathogenetic effects of endogenous AhR are unclear, but research indicates that the RelB protein (encoded by a target gene of NF-κB) may be partially responsible: AhR is known to interact with RelB and to modulate its expression [290]. Moreover, AhR regulates oxidative stress. When exposed to cigarette smoke, AhR-deficient lung cells show a higher production of reactive oxygen species and a lower expression of antioxidant enzymes (NQO1 and sulfiredoxin) than do lung cells with normal AhR levels, suggesting that cigarette smoke-induced oxidative stress is enhanced in AhR-deficient lungs [291].

AhR activation can influence the inflammatory phase in both asthma and chronic obstructive pulmonary disease through inflammatory and resident cells in the lungs [282,284]. The relation between AhR function and airway inflammation in the initial phase is important not only in chronic obstructive pulmonary disease but also in asthma. Airway Clara cells are sensitive to AhR activation by the ligand TCDD, and these cells are capable of secreting a wide range of glycoproteins such as mucins and SP-D [274,292]. TCDD upregulates inflammatory cytokines, COX2, MUC5AC, and MMPs via AhR signaling in a Clara cell-derived cell line. Research articles about AhR agonists and inhibitors have shown that AhR activation induces the production of such cytokines as TGF-α and tumor necrosis factor and of MMPs through receptors in human hematocytes and epithelial cells [282,292,293,294]. Thus, typical AhR xenobiotic ligands such as TCDD and BaP may contribute to the development of lung diseases. 

Nevertheless, there are observations strongly indicating that AhR signaling can be beneficial in lung diseases mediated by inflammation and oxidative damage [295]. A deficiency of AhR signaling affects immune and nonimmune cells such as neutrophils, macrophages, and fibroblasts in the lungs, thereby resulting in greater pulmonary inflammation after exposure to tobacco smoke, lipopolysaccharide, and hyperoxia [296,297]. Conversely, AhR activation is known to reduce airway inflammation in rodent models of asthma by modulating the production and secretion of Th2 cytokines such as interleukin (IL) 4, IL-5, and IL-15 [298,299]. The activation of AhR by omeprazole alleviates lung inflammation in a model of acute hyperoxic lung injury based on adult mice, while neutrophil infiltration and MCP-1 expression are weaker as compared to vehicle-treated animals [300].

Because AhR takes part in the initiation of the aforementioned lung diseases, the development of biologics and small-molecule compounds that target the AhR signaling pathway is a possible approach to the prevention and treatment of these pathologies. For instance, AhR antagonist resveratrol has been shown to diminish mucin production [279]. AhR antagonist CH223191 attenuates BaP-induced allergic lung inflammation via AhR [301]. This AhR antagonist has also been reported to reverse experimental pulmonary hypertension induced by Sugen 5146 in rats [302].

### 5.4. Rheumatoid Arthritis 

This is an autoimmune disease characterized by the chronic inflammation of the joints and the tissues around the joints along with infiltration by macrophages and activated T cells [126,303]. The pathogenesis of this disease is due to the formation of reactive oxygen and nitrogen species at the site of inflammation. The oxidative damage and inflammation have been confirmed in various rheumatic diseases by means of elevated levels of isoprostane and prostaglandins in serum and synovial fluid as compared with a control group [304]. Environmental factors, especially polycyclic aromatic hydrocarbons, are implicated in the pathogenesis of rheumatoid arthritis; hence, the role of AhR in this pathogenesis is of interest. Many papers indicate that polycyclic aromatic hydrocarbons play a critical part in the development of rheumatoid arthritis in various ways [305,306,307], primarily by influencing changes in the diversity of immune cells and the related downstream cytokines, and the main route of these effects goes through the AhR signaling pathway.

AhR, a major immunomodulator, is expressed in various cells (T and B cells, natural killers, macrophages, and dendritic cells) involved in rheumatoid arthritis [308]. Of note, in different cells implicated in rheumatoid arthritis, the effects of AhR activation are opposite. For instance, in Th1 and Th17 cells, B cells, dendritic cells, M1 monocytes, natural killers, and osteoclasts, AhR activation has an exacerbating impact on rheumatoid arthritis [309,310,311,312,313,314]; however, in Th2 cells, regulatory T cells, regulatory dendritic cells, M2 monocytes, and osteoblasts, AhR activation protects against rheumatoid arthritis [311,314,315,316]. 

AhR ligands such as 6-formylindolo[3,2-*b*]carbazole, TCDD and BaP relieve experimental arthritis; nonetheless, the long-term administration of these compounds has serious adverse effects such as high embryonic mortality, hepatotoxicity, and carcinogenicity [317,318,319], which limit the use of these compounds as therapeutic agents in animals or humans. AhR agonists with fewer adverse effects may be candidate therapeutics for rheumatoid arthritis [308]. Although much basic research has been conducted on the role of polycyclic aromatic hydrocarbon-activated AhR in rheumatoid arthritis, clinical studies on its effects on the mechanism of AhR signaling in rheumatoid arthritis are still lacking, and further research is needed.

### 5.5. Skin Diseases

These diseases may also be linked with environmental pollutants having high affinity for AhR [84,320]. Low doses of these compounds cause skin irritation or worsen symptoms of diseases [321]. Exposure to high doses of air pollutants leads to such skin pathologies as chloracne and hyperpigmentation [322,323,324,325]. AhR participates in many pathological processes in the skin through alterations in the signaling pathways controlled by AhR.

Early studies pointed to a partial role of AhR in the pathogenesis of various skin diseases, including inflammatory diseases, skin pigmentation disorders, and skin cancer [131,179,326], as well as a dependence of the outcome of AhR activation on the cell type and ligand [130,327]. Several papers offer evidence of AhR’s involvement in the pathogenesis of chloracne, hyperpigmentation, and vitiligo, as well as inflammatory diseases such as psoriasis and atopic dermatitis [116,328,329,330,331]. Additionally, many different biological responses to AhR stimulation or inhibition are observed in the skin [177].

On the one hand, the activation of AhR by ligands can induce the overexpression of proinflammatory cytokines and the production of reactive oxygen species, yielding an inflammatory disease or carcinogenesis [173]. On the other hand, AhR activity can influence the differentiation of regulatory T cells, thereby promoting immune tolerance [332]. A large class of tryptophan derivatives that are AhR ligands may play a part in the pathogenesis or treatment of many skin diseases [73,333]. Tryptophan derivatives are generated by enzymatic reactions or by exposure to ultraviolet light in various skin cells, and some of these compounds are present in herbs and plant extracts commonly used for skin care and therapies. Their biological activities require further research [100,334].

### 5.6. Nephropathies 

Oxidative stress participates in various renal pathologies such as glomerulonephritis, tubulointerstitial nephritis, proteinuria, uremia, diabetic nephropathy, and chronic renal failure [335]. In chronic kidney disease, especially with uremia, oxidative stress can be explained by high pro-oxidant activity driving the overproduction of reactive oxygen species. The reasons for this phenomenon are complicated and multifactorial, and are mostly linked with elevated NOX activity, markedly upregulated xanthine oxidase, and concomitant mitochondrial dysfunction [336].

The roles of oxidative stress and inflammation in kidney failure lie in their influence on the most common disorders accompanying chronic kidney disease, which progress further via a positive-feedback mechanism, promoting an additional enhancement of oxidative stress and the progression of chronic kidney disease with a full range of complications [336]. Many authors have reported that AhR is associated with chronic kidney disease and its complications. A review of current knowledge on the participation of AhR in chronic kidney disease [337] shows that AhR mediates chronic kidney disease complications, including cardiovascular disorders, anemia, bone disorders, cognitive dysfunction, and malnutrition, and that AhR affects drug metabolism in patients with chronic kidney disease [337]. 

AhR also helps to reduce the harmful effects of uremic toxins by strengthening intestinal-barrier function [337,338]. In patients with chronic kidney disease, the key AhR ligands are the uremic toxins that arise during the metabolism of tryptophan, which is a precursor of a large number of microbial and host metabolites [338]. Derivatives of kynurenine, of serotonin, and of indole are (by-)products of the three main tryptophan metabolic pathways, which are directly or indirectly modulated by the gut microbiota [338]. The tryptophan metabolites indoxyl sulfate, indole-3-acetic acid, and kynurenine are not only important uremic toxins but are also potent AhR ligands [339,340,341]. Activated AhR is proven to exacerbate kidney damage [342,343,344]. An increase in AhR activity—in the periglomerular region and in proximal and distal renal tubules—under the action of adenine and indoxyl sulfate leads to renal fibrosis [345]. Indoxyl sulfate-activated AhR causes podocyte injury, progressive glomerular damage, and a proinflammatory phenotype [346]. Chronic kidney disease may be aggravated by indoxyl sulfate via the OAT3–AhR–STAT3 cascade in proximal tubular cells owing to the downregulation of a receptor called MAS, the subsequent inhibition of the renin–angiotensin system, and TGF-β activation [347]. Renal fibrosis may be mediated by AhR signaling [338,348].

AhR activity in patients with diabetes mellitus positively correlates with the progression of diabetic nephropathy and kidney failure severity [349]. A paper about the function of AhR in the pathophysiological processes of diabetic nephropathy (on the basis of an AhR knockout mouse model and a pharmacological inhibitor, α-naphthoflavone) has revealed that AhR mediates renal oxidative stress in a diabetic mouse model, thereby inducing infiltration by macrophages, extracellular matrix accumulation, and mesangial cell activation [350].

### 5.7. Cardiovascular Diseases

These diseases have a multifactorial etiology related to various risk factors, including but not limited to hypercholesterolemia, hypertension, tobacco smoking, diabetes mellitus, poor diet, stress, and lack of physical activity. Evidence has been published supporting the participation of oxidative stress in a number of cardiovascular disorders such as atherosclerosis, ischemia, hypertension, cardiomyopathy, cardiac hypertrophy, and congestive heart failure [351,352,353,354]. 

AhR is closely connected with cardiovascular diseases in terms of cardiac function, vascular development, and blood pressure regulation. In some diseases associated with atherosclerosis, AhR can serve as a transmitter of an oxidative stress signal [62,355,356]. 

There is a strong association between the accumulation of uremic toxic compounds and cardiovascular complications of chronic kidney disease [357]. AhR activation directly correlates with cardiovascular risk, even in the absence of uremia [358,359]. People exposed to AhR agonists are at an increased cardiovascular risk. To give an example, people who were exposed to TCDD as part of Agent Orange during the Vietnam War were more likely to experience relevant cardiovascular complications such as coronary heart disease and stroke [360]. 

Environmental pollutants that are AhR ligands promote the onset and progression of atherosclerosis, indicating that AhR may partake in the regulation of atherosclerosis [361,362,363]. Inflammatory responses contribute to AhR-regulated atherosclerosis. There are three hypotheses based on the AhR signaling pathways that mediate inflammation and promote atherosclerosis. The first hypothesis involves downstream inflammatory signaling factors such as VCAM-1 acting through the AhR–NF-κB signaling pathway, resulting in monocyte chemotaxis. Macrophages and monocytes are targets of polycyclic aromatic hydrocarbons, which are implicated in the physiological and pathological processes of atherosclerosis [364,365]. The second hypothesis postulates that AhR promotes absorption of oxidized low-density lipoprotein by macrophages giving rise to foam cells with the help of endogenous and exogenous ligands such as oxidized low-density lipoprotein, lipopolysaccharides, and TCDD. In vitro experiments have revealed that cholesterol accumulation in foam cells under the influence of particulate matter-induced inflammation is an early sign of cardiovascular diseases. Nonetheless, the suppressive action of AhR inhibitors on foam cells and on inflammation has not been investigated. It is believed that these mechanisms will be clarified by extensive studies on AhR [366,367]. The third hypothesis is the accelerated proliferation of vascular smooth muscle cells, which is key to the initiation of vascular complications according to the finding that indoxyl sulfate induces the proliferation of vascular smooth muscle cells through AhR activation, NF-κB signal transduction, and the production of reactive oxygen species [368]. It should be said that the division of the pathological involvement of AhR in the development of atherosclerosis into three hypotheses is largely arbitrary. These processes are related, and this division can only reflect the structuring and emphasis in future studies that should clarify the activity of this complex molecular network.

The regulation of AhR at the *AhR* transcription level in humans has not been elucidated yet. AhR is connected with other signaling pathways, including Wnt and E2 cascades, and further research is needed to clarify the function of AhR and identify new endogenous ligands in order to elucidate the role, regulation, and possible usefulness of AhR in the treatment of atherosclerosis. AhR is reported to be a major participant in the pathogenesis of such cardiovascular pathologies as myocarditis, hypertension, coronary heart disease, and pulmonary arterial hypertension [62]. The pathogenesis driven by AhR varies among cardiovascular diseases, but includes inflammatory responses, immune responses, oxidative stress, and endothelial dysfunction. The molecular mechanisms behind AhR signaling and behind the crosstalk between AhR signaling and other signal transduction cascades still require further investigation [62].

## 6. Conclusions

**Figure 4:**
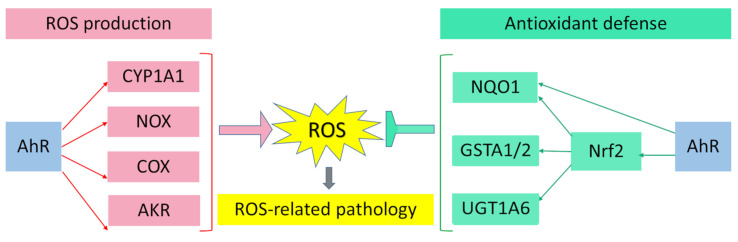
Pro-oxidant and antioxidant effects of AhR results in wide range of physiological and pathological processes in cells and tissues.

Major breakthroughs were recently made in the biology of redox modulation by AhR. Despite all the gained knowledge, the remaining intriguing questions concern the mechanism underlying the cell- and tissue-specific effects of AhR ligands and the dependence of responses on the type of ligands. The function of AhR is complicated because the outcome of its activation depends on a wide range of endogenous and exogenous ligands (which are characterized by different affinity values and diverse combinatorial effects) and on different AhR functions in many physiological and pathological processes in cells and tissues. The molecular mechanisms of AhR signaling and of the crosstalk between AhR signaling and other signal transduction cascades require further research. It is mostly the inconsistency of scientific findings that makes it difficult to determine the signaling pathways through which AhR can exert its beneficial or detrimental actions. There is growing evidence that AhR activation can have multidirectional effects on many aspects of human physiology and pathology, and that these may depend on cell and tissue types, or on the interaction of the AhR complex with non-traditional XRE sequences, or interaction with various coactivators and corepressors. Depending on many factors, the action of AhR agonists or antagonists can cause positive or negative effects on human health (Figure 4).

The recognition that AhR is implicated in the pathogenesis of many human diseases has arisen in conjunction with numerous examples of diseases in which AhR modulates disease activity through interaction with environmental factors. The pathogenesis driven by AhR often includes oxidative stress and immune and inflammatory responses. The weight of evidence indicates that, in diseases of various organs and tissues, AhR activation can be beneficial or detrimental. The ultimate effect depends both on the context of the disease and on the nature of AhR ligands. In this context, AhR activation aggravates the symptoms of some diseases, but alleviates the symptoms of other diseases.

Currently, in the literature, there are few examples of disorders where the molecular mechanisms of AhR’s involvement in the pathogenesis are clear. More numerous are findings about various biological responses to the stimulation or inhibition of AhR in various diseases. At the current stage of our insight into AhR’s biology and its role in the pathogenesis of diverse diseases, the utility of AhR as a therapeutic target has already been established, and a foundation has been laid for the selection and design of effective AhR ligands as new treatments of various diseases. Although much basic research has been conducted on the functions of AhR in pathological processes, clinical studies about the effects on the mechanism of the AhR signaling pathway in different pathologies are still scarce, and further investigation is necessary.

## Figures and Tables

**Table 1:** The list of AhR ligands.

Exogenous Compounds		Endogenous Compounds
Synthetic compounds	Natural Compounds	Indigoids
Polycyclic aromatic hydrocarbons	Polyphenols	Eicosanoids
Polychlorinated biphenyls	Diosmin	Tryptophan metabolites:
Halogeneted Dioxins and Related Compounds	Resveratrol	L-Kynurenine,
Other synthetic AhR Ligands:	Curcumin	Kynurenic acid,
Benzimidazole	Berberin	
Pesticides	Alcaloids	*ITE*—(2-(1′H-indole-3′-carbonyl)-thiazole-4-carboxylic acid methyl ester,
Primaquine	(Tetrandrine,	Indoxyl-3-sulfate,
Kinase inhibitor	Sinomenine Norisoboldin)	Indirubin,
Synthetic flavonoid	Dietary compounds (Indole-3-carbinol Indole-3-acetonitrile,	Tryptamine,
New synthetic ligands	3,3′-Diindolylmethane Indolo(3,4)bicarbazole)	3-Methylindole
(CH223191, VAF347, 4OHT,		Ultraviolet photoproducts of tryptophan:
6-MCDF)		FICZ—6-Formyl indolo (3,2-b) carbazole
		Heme metabolites
		Bilirubin
		Biliverdin
		Arachidonic acid metabolites:
		12(R)-hydroxy-5(Z),8(Z),10(E)
		14(Z)-eicosatetraenoic acid

## Data Availability

Not applicable.

## Abbreviations

AhR	aryl hydrocarbon receptor
AhRR	aryl hydrocarbon receptor repressor
AKR	aldo–keto reductase
ARE	antioxidant response element
ARNT	aryl hydrocarbon receptor nuclear translocator
BAFF	B-cell-activating factor of the tumor necrosis factor family
BaP	benzo[a]pyrene
bHLH	basic helix–loop–helix
BLC	B-lymphocyte chemoattractant
CCL1	CC-chemokine ligand 1
COX	cyclooxygenase
CYP	cytochrome P450
E2F1	E2 promoter binding factor 1
EGF	epidermal growth factor
GPx	glutathione peroxidase
GST	glutathione S-transferase
HSP90	heat shock protein 90
IDO1	indolamine-2,3-dioxygenase 1
IFR3	interferon responsive factor
IL	interleukin
Keap1	Kelch-like ECH-associated protein 1
KLF6	Kruppel-like Factor 6
MAPK	mitogen-activated protein kinase
MMP	matrix metalloproteinase
NADPH	nicotinamide adenine dinucleotide phosphate
NF-κB	nuclear factor κB
NOX	NADPH oxidase
NQO1	NADPH:quinone oxidoreductase-1
Nrf2	nuclear factor erythroid 2-related factor 2
PAS	Per–ARNT–Sim
pRB	retinoblastoma protein
TAD	transactivation domain
TCDD	2,3,7,8-tetrachlorodibenzo-p-dioxin
TDP-43	TAR DNA-binding protein 43 kDa
TGF	transforming growth factor
UGT	UDP glucuronosyltransferase
VEGF	vascular endothelial growth factor
XRE	xenobiotic-responsive element
XREL1	XRE-like element
